# TSG-6 Inhibits the NF-κB Signaling Pathway and Promotes the Odontogenic Differentiation of Dental Pulp Stem Cells via CD44 in an Inflammatory Environment

**DOI:** 10.3390/biom14030368

**Published:** 2024-03-19

**Authors:** Ying Wang, Yulang Xie, Ningning Xue, Hao Xu, Dunfang Zhang, Ning Ji, Qianming Chen

**Affiliations:** 1 State Key Laboratory of Oral Diseases, National Clinical Research Center for Oral Diseases, Research Unit of Oral Carcinogenesis and Management, Chinese Academy of Medical Sciences, West China Hospital of Stomatology, Sichuan University, Chengdu 610041, China; 2 State Key Laboratory of Biotherapy and Cancer Center, Department of Biotherapy, Collaborative Innovation Center of Biotherapy, West China Hospital, Sichuan University, Chengdu 610041, China

**Keywords:** NF-κB signal pathway, dental pulp stem cells, inflammation, tumor necrosis factor-inducible protein 6

## Abstract

In pulpitis, dentinal restorative processes are considerably associated with undifferentiated mesenchymal cells in the pulp. This study aimed to investigate strategies to improve the odonto/osteogenic differentiation of dental pulp stem cells (DPSCs) in an inflammatory environment. After pretreatment of DPSCs with 20 ng/mL tumor necrosis factor-induced protein-6 (TSG-6), DPSCs were cultured in an inflammation-inducing solution. Real-time polymerase chain reaction and Western blotting were performed to measure the expression levels of nuclear factor kappa B (NF-κB) and odonto/osteogenic differentiation markers, respectively. Cell Counting Kit-8 and 5-ethynyl-2′-deoxyuridine assays were used to assess cell proliferation and activity. Subcutaneous ectopic osteogenesis and mandibular bone cultures were performed to assess the effects of TSG-6 in vivo. The expression levels of odonto/osteogenic markers were higher in TSG-6-pre-treated DPSCs than nontreated DPSCs, whereas NF-κB-related proteins were lower after the induction of inflammation. An anti-CD44 antibody counteracted the rescue effect of TSG-6 on DPSC activity and mineralization in an inflammatory environment. Exogenous administration of TSG-6 enhanced the anti-inflammatory properties of DPSCs and partially restored their mineralization function by inhibiting NF-κB signaling. The mechanism of action of TSG-6 was attributed to its interaction with CD44. These findings reveal novel mechanisms by which DPSCs counter inflammation and provide a basis for the treatment of pulpitis.

## 1. Background

Pulpitis is a type of inflammation of the loose connective tissue inside the hard tissue of the teeth. Pulpitis is caused by decay, trauma, or other stimulating factors, and is usually accompanied by pain and a deficiency of dental tissue [1,2,3]. The main treatment for irreversible pulpitis is root canal therapy [4]. However, the loss of vascular nerve tissue in the teeth causes a lack of nutritional supply, and the hard tissue is easily fractured and discolored [5]. Dental pulp stem cells (DPSCs) are present in the pulp tissue and can proliferate and differentiate into odontoblasts to repair defects when stimulated by an external source [6]. 

DPSCs are derived from ectodermal mesenchyme and can undergo multidirectional differentiation [7]. Extensive research has been conducted on the use of DPSCs for treating immune diseases and combining them with materials to repair tissue defects [8]. In addition, inflammation could cause oxidative and metabolic disorders to impair the function of mesenchymal stem cells [9]. In vitro, low concentrations of inflammatory factors could promote the proliferation and differentiation of DPSCs, but high concentrations of them could cause a decrease in the mineralization ability of DPSCs [10]. This is consistent with the pathological process of pulpitis: if pulpitis is extensively aggressive and exceeds the damage repair potential of the DPSCs, dentin destruction becomes extremely rapid, exceeding the DPSCs’ repair capacity [1]. This can result in severe tooth deterioration, pulpal inflammation, and pulpal exposure. DPSCs can be used for the restorative treatment of pulpitis. Therefore, improving DPSCs’ repair capacity under inflammatory conditions is a promising treatment strategy.

Tooth development is associated with many signaling pathways, including the Wnt, Notch, and bone morphogenetic protein (BMP) signaling pathways [11]. The nuclear factor kappa B (NF-κB) pathway is a molecular pathway classically associated with inflammation and related to tissue mineralization, and NF-κB shows high expression levels in inflamed pulp tissue [12]. Similar to mesenchymal stem cells (MSCs), DPSCs express NF-κB-signaling-pathway-related molecules in an inflammatory environment [13]. Activation of the NF-κB signaling pathway also inhibits the mineralization capacity of DPSCs [14]. Exogenous supplementation with tumor-necrosis-factor-induced protein-6 (TSG-6) can inhibit the NF-κB signaling pathway through CD44 on the cell membrane [15].

TSG-6 is mainly expressed by MSCs and stimulated by tumor necrosis factor-alpha (TNF-α) and interleukin 1 (IL-1) to fight inflammation [16]. TSG-6 is an important component of stem cell therapy. The anti-inflammatory properties of TSG-6 can be exploited to treat autoimmune diseases, such as systemic lupus erythematosus and peritonitis [17,18]. The expression level of TSG-6 decreases with the differentiation of MSCs [19]. In addition, TSG-6 affects mineralization-related diseases. The anti-inflammatory activity of TSG-6 is thought to underlie its cartilage-protective effects in rheumatoid arthritis (RA) models, and the application of TSG-6 protein or its overexpression significantly attenuates joint damage in RA models [20,21]. 

Furthermore, TSG-6 reduces cytokine expression by downregulating the NF-κB signaling pathway and promoting macrophage polarization toward the M2 (anti-inflammatory) phenotype [22]. TSG-6 interacts with different BMPs and RANKL, affecting osteogenesis and bone destruction. TSG-6 inhibits the BMP-2-mediated osteoblast differentiation of MSCs [23]. A previous study reported that the bone density of TSG-6/− mice was notably higher than that of the control mice; this suggests that TSG-6 plays a role in bone homeostasis [24]. TSG-6 exerts different effects on mineralization under different conditions. 

Thus, TSG-6 appears to inhibit odontogenic bone resorption. Overexpression of TSG-6 in rat iPSC-derived MSCs inhibits inflammation in experimental periodontitis and decreases alveolar bone resorption [25]. However, whether exogenous supplementation with TSG-6 rescues the odonto/osteogenic differentiation of DPSCs in an inflammatory environment has not yet been investigated. The cell membranes of MSCs stably express CD44, suggesting that exogenous TSG-6 may also act on DPSCs. 

This study aimed to explore approaches aimed at protecting dental pulp tissue from inflammation and restoring the mineralization capacity of DPSCs in an inflammatory environment. The findings of this study contribute to the development of new strategies for the treatment of pulpitis.

## 2. Methods

### 2.1. Cell Culture and Identification

Human DPSCs were isolated from the third molars of healthy patients aged 18–22 years, without caries, cysts, or periodontal and periapical diseases. Informed consent was obtained from all patients. The pulp tissue samples were placed in Dulbecco’s modified Eagle’s medium (DMEM; HyClone Laboratories, Logan, UT, USA) containing 10% penicillin–streptomycin (Solarbio, Beijing, China) for 1 h. They were then rinsed three times with phosphate-buffered saline (PBS; Solarbio). The pulp tissue was removed from the tooth crown using a clean pulp extraction needle and placed in PBS containing 1% penicillin–streptomycin. The removed pulp tissue was cut into 1 mm × 1 mm × 1 mm blocks. The tissues were digested with 3 mg/mL type I collagenase (Invitrogen, Waltham, MA, USA) for 40 min at 37 °C. The resulting DPSCs were cultured in DMEM supplemented with 20% fetal bovine serum (FBS; Gibco, Billings, MT, USA), and the solution was changed every 2 days. After culturing, the fusion rate was 80%, and cell passages were performed. P3-generation DPSCs were used in the experiments.

Osteogenesis induction [26]: DPSCs were inoculated into six-well plates and induced with an osteogenic-inducing solution (10 mmol/L β-glycerophosphate; Sigma-Aldrich, St. Louis, MO, USA; 10^−8^ mol/L dexamethasone, Sigma Aldrich; 50 μg/mL ascorbic acid, Sigma Aldrich; 0.01 μmol/L 1,25-dihydroxy vitamin D3, Solarbio) when they reached a 70% fusion rate. After 21 days, the DPSCs were fixed with 4% paraformaldehyde (Solarbio), stained with 0.1% chymotrypsin (Solarbio), and washed three times. The mineralized nodules were observed under a light microscope.

Adipogenesis induction [26]: The pretreatment of the cells was the same as that used for osteogenesis induction. DPSCs were cultured in lipogenic medium (Cyagen Biosciences, Suzhou, China) for 18 days, fixed with 4% paraformaldehyde, and stained with 0.3% Oil Red O. Lipid droplet formation was observed under a light microscope.

Flow cytometry analysis: DPSCs were digested with trypsin into single-cell suspensions and incubated with phycoerythrin-conjugated CD3 (1:100 dilution), CD34 (1:100 dilution), or CD31 (1:100 dilution); fluorescein isothiocyanate (FITC)-conjugated CD90 (1:100 dilution); or allophycocyanin-conjugated CD73 (1:200 dilution) antibodies. These suspensions were then used to determine the expression levels of cell-surface molecules. All antibodies were purchased from BD Biosciences (San Jose, CA, USA). Flow cytometry was performed using a fluorescence-activated cell sorting flow cytometer (BD Biosciences).

Cell-surface molecule identification: DPSCs were inoculated into six-well plates and fixed with 4% paraformaldehyde for 20 min. The cells were then permeabilized in 0.1% Triton-100 (Solarbio) for 10 min, washed thrice with PBS, and washed with 10% goat serum (Solarbio). They were blocked at 37 °C for 1 h and then incubated with primary antibodies (anti-vimentin, 1:200 dilution, Abcam, Cambridge, UK; anti-cytokeratin 14, 1:200 dilution, Millipore Sigma, St. Louis, MO, USA; anti-STRO-1, 1:100 dilution, Santa Cruz Biotechnology, Dallas, TX, USA) overnight at 4 °C. This was followed by incubation with secondary antibodies (cy-3-conjugated goat anti-mouse, 1:500 dilution; Beyotime Biotechnology Co., Ltd., Shanghai, China; FITC-conjugated goat anti-rabbit, 1:500 dilution; Beyotime Biotechnology) for 1 h at room temperature in the dark. Finally, 4’,6-diamidino-2-phenylindole (DAPI; Solarbio) was added for 10 min at room temperature to stain the cell nuclei.

### 2.2. Cell Induction

Cells in the normal group were incubated in normal complete medium (DMEM containing 5% FBS). Cells in the odonto/osteogenic-induction group were cultured with edentulous odonto/osteogenic-inducing solution (mineralization-inducing solution) comprising 5% FBS, 10 mmol/L sodium β-glycerophosphate, 10^−7^ mol/L dexamethasone, and 50 ng/mL ascorbic acid. Cells in the inflammation group (TNF-α group) were incubated with a mineralization-inducing solution containing 50 ng/mL TNF-α. Cells in the TSG-6 group were incubated in normal complete medium containing 20 ng/mL TSG-6 (R&D Systems Inc., Minneapolis, MN, USA). The cells were incubated for 48 h and then transferred to an inflammation-inducing solution. Cells in the neutralizing antibody group (anti-CD44 group) were incubated with TSG-6 and an anti-CD44 antibody (Bio X Cell, Lebanon, NH, USA) for 48 h, and then transferred to an inflammation-inducing solution.

### 2.3. Real-Time Polymerase Chain Reaction

DPSCs were cultured in normal medium, mineralization-inducing solution, inflammation-inducing solution, or TSG-6-inducing solution for 7 days. Total mRNA was extracted using TRIzol reagent (Takara, Shiga, Japan). Reverse transcription was performed by Takara Bio (Shiga, Japan). cDNA was amplified using a fluorescent quantitative polymerase chain reaction (PCR) kit (Takara) and an Applied Biosystems 7300 Real-Time PCR System (Applied Biosystems, Foster City, CA, USA). *GAPDH*, *ALP*, *RUNX2*, *DSPP*, and *DMP-1* expression levels were determined. The primer sequences were obtained from the literature [19,27,28]. The PCR program was as follows: 95 °C for 30 s; 40 cycles of 95 °C for 5 s and 60 °C for 34 s; 95 °C for 15 s; and 60 °C for 1 min. All experimental steps were performed according to the manufacturer’s instructions, and the relative expression levels were calculated using the 2^−ΔΔCt^ method.

### 2.4. P65 Nuclear Transfer Assay

DPSCs were cultured in normal medium, mineralization-inducing solution, inflammation-inducing solution, or TSG-6-inducing solution for 48 h. DPSCs were fixed with 4% paraformaldehyde for 30 min and cell permeability was promoted using 0.1% TritonX-100. Each group of DPSCs was washed thrice in PBS and blocked with 5% BSA at room temperature for 1 h, followed by incubation with the primary and secondary antibodies. All procedures were performed using a reagent kit (Beyotime Biotechnology). Finally, DAPI was applied and the cells were observed under an inverted fluorescence microscope.

### 2.5. Cell Counting Kit-8

Each group of cells was cultured for seven days with several induction solutions, and the optical density (OD) was measured on days 1, 3, 5, and 7. Cell Counting Kit-8 (CCK-8) reagent (Beyotime Biotechnology) was prepared at a concentration of 10% prior to the assay. DMEM alone was used to prepare the CCK-8 assay solution. The cells were incubated in CCK-8 assay solution at 37 °C for 2 h in the dark. The OD values were recorded at 450 nm.

### 2.6. Determination of Alkaline Phosphatase Activity

DPSCs were inoculated into six-well plates and cultured for 3 days. Alkaline phosphatase (ALP) assay reagent (Beyotime Biotechnology) was prepared according to the manufacturer’s protocol. The cells were observed under an inverted fluorescence microscope. Quantitative analysis was performed using ImageJ software (Institutes of Health, Bethesda, MD, USA).

### 2.7. 5-Ethynyl-2’-deoxyuridine Cell Activity Assay

DPSCs were induced for 48 h. A 5-ethynyl-2’-deoxyuridine (EdU) staining solution (RiboBio Co., Ltd., Guangzhou, China) was diluted 1:1000 in complete medium. After induction with the staining solution, the DPSCs were cultured at 37 °C for 2 h, washed three times with PBS, and observed under a fluorescence microscope.

### 2.8. Western Blotting Analysis

Total protein was extracted using radioimmunoprecipitation assay lysis buffer (Beyotime Biotechnology) containing phosphatase inhibitors. Nuclear and plastid proteins were extracted using a Nuclear Protein Extraction Kit (SolarBio). The expression levels of NF-κB-signaling-pathway-related proteins, including the nuclear proteins p65 and phospho-p65 (p-p65) and the plastid proteins p-IκB and IκB, were measured after 48 h of induction. In addition, the expression levels of odonto/osteogenesis-related proteins, including DMP-1, DSPP, and RUNX2, were determined after seven days. Proteins in each group were separated on polyacrylamide gels, transferred to polyvinylidene fluoride membranes (Millipore Sigma), and blocked with 5% BSA (Solarbio) at room temperature for 2 h. The membranes were then incubated overnight at 4 °C with primary antibodies, including anti-p65 (1:1000 dilution; Cell Signaling Technology, Danvers, MA, USA), anti-p-p65 (1:2000 dilution; Abcam, Cambridge, UK), anti-p-IκB (1:1000 dilution; Cell Signaling Technology), anti-IκB (1:1000 dilution; Cell Signaling Technology), anti-DSPP (1:1000 dilution; Bioworld, Nanjing, China), anti-DMP-1 (1:100 dilution; Thermo Fisher Scientific, Waltham, MA, USA), anti-RUNX2 (1:1000 dilution; Abway, Beijing, China), anti-GAPDH (1:10,000 dilution; ZenBio, Chengdu, China), and anti-lamin B1 (1:1000 dilution; Santa Cruz Biotechnology). Membranes were then incubated with horseradish-peroxidase-conjugated anti-rabbit or anti-mouse IgG secondary antibodies (Biyuntian Biotechnology Co., Ltd., Shanghai, China) at room temperature for 1 h. The labeled proteins were visualized using an Imager 600 instrument (GE Amersham, Amersham, UK), and ImageJ software was used to quantify the results. Protein levels were quantified relative to GAPDH or lamin B1 levels.

### 2.9. Subcutaneous Transplantation

The animal experiments were approved by the Ethics Committee of West China Hospital, Sichuan University. The animals were divided into three groups to compare the effects of exogenous TSG-6 on the odonto/osteogenesis of DPSCs in an inflammatory environment. DPSCs were cultured in mineralization-inducing solution or inflammation-inducing solution for seven days. TSG-6-pretreatment DPSCs were cultured in inflammation-inducing solution for seven days. The cells and culture solutions were then mixed with Corning PuraMatrix peptide hydrogel (Corning Life Sciences, Corning, NY, USA) and injected into the backs of immunodeficient mice (BALB/c-nu; 6-week-old, males, n ≥ 3 per group) at a density of 1 × 10^6^ cells/injection. The volume ratio of cell culture medium to hydrogel was 2:1. The mixtures were left at room temperature for 5 min and then injected subcutaneously into the nude mice. The immunodeficient mice were purchased from Chengdu Collective Pharmacology Biotechnology Co., Ltd. (Chengdu, China). After six weeks, subcutaneous tissue samples were collected, fixed overnight in 4% paraformaldehyde, and embedded in paraffin wax. Paraffin sections were prepared and stained with hematoxylin and eosin (HE), Masson’s trichrome, and immunohistochemical stains. Histological staining (HE and Masson’s) and immunohistochemical staining methods refer to the previous literature [29].

The antibodies used for immunohistochemistry were anti-DMP-1 (1:100 dilution), anti-DSPP (1:100), anti-COL-1 (1:300 dilution; Wuhan Servicebio Technology, Co., Ltd., Wuhan, China), and anti-RUNX2 (1:500 dilution) antibodies. Secondary antibodies were visualized using a 3,3′-diaminobenzidine color development kit (Zhongshan Jinqiao Biotechnology Co., Ltd., Beijing, China). Positive signals from the immunohistochemical images were measured using ImageJ software.

### 2.10. Rat Mandibular Bone Culture

Two-day-old rats were sacrificed under isoflurane anesthesia and their mandibles were removed. Gelatin sponges were placed in six-well plates and the mandibles were cultured for 10 days in a solution containing dimethyl sulfoxide, mineralization-inducing solution, inflammation-inducing solution, or TSG-6-inducing solution (the TSG-6 group was treated with normal medium containing 20 ng/mL TSG-6 for 48 h and then placed in the inflammation-inducing solution). They were fixed in 4% paraformaldehyde and demineralized using EDTA. HE and Masson’s trichrome staining were used to evaluate the mineralization capacity of each group.

### 2.11. Statistical Analysis

All data are expressed as the mean ± standard deviation. Statistical differences were analyzed using SPSS version 21.0 software (IBM Corp., Armonk, NY, USA). Corrected paired Student’s *t*-tests and one-way analysis of variance with Tukey’s post hoc test were used to calculate the level of significance. Statistical significance was set at *p* < 0.05.

## 3. Results

### 3.1. Isolation, Culture, and Identification of DPSCs

**Figure 1:**
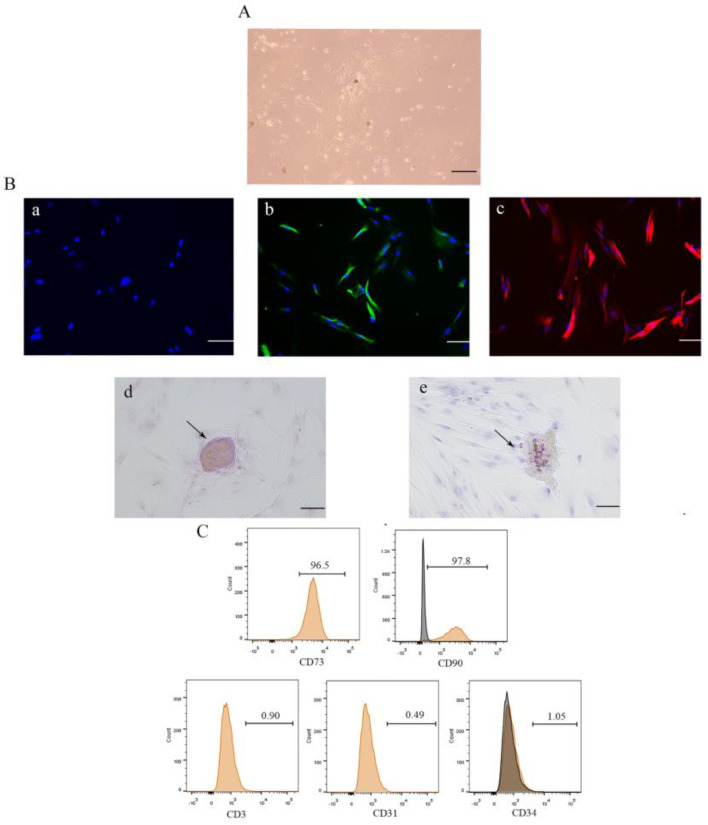
Culture and identification of dental pulp stem cells. Isolated cells were subjected to marker identification, multidirectional induction, and surface molecule identification. (**A**) Cell morphology was observed by light microscopy. (**B**) Cellular immunofluorescence analysis was performed to determine CK-14 (**Ba**), vimentin (**Bb**), and STRO-1 (**Bc**) protein expression levels. (**Bd**,**Be**) Mineralized nodules and lipid droplets were observed after 21 days of osteogenic induction and after 18 days of lipogenic induction. (**C**) Flow cytometry was performed to observe mesenchymal stem cell (MSC) surface markers. Scale bars = 20 μm.

DPSCs were observed under a light microscope (Figure 1A). These cells did not express the epithelial marker CK-14 (Figure 1(Ba)), indicating that they were not epithelial cells. Immunofluorescence staining showed that the cells expressed vimentin and STRO-1 (Figure 1(Bb,Bc)), indicating that they were of mesenchymal origin. The cells produced mineralized nodules 21 days after osteogenic induction (Figure 1(Bd)) and lipid droplets 18 days after lipogenic induction (Figure 1(Be)), indicating that they had multidirectional differentiation potential and stem cell properties. In addition, flow cytometry showed that the cells expressed MSC surface markers, including CD73 and CD90, but not endothelial cell surface markers, such as CD31 and CD34, or the T cell surface marker CD3 (Figure 1C). These results suggested that the cells were DPSCs.

#### 3.1.1. Exogenous TSG-6 Inhibited the NF-κB Signaling Pathway and Partially Rescued the Suppressed Mineralization of DPSCs in an Inflammatory Environment

**Figure 2:**
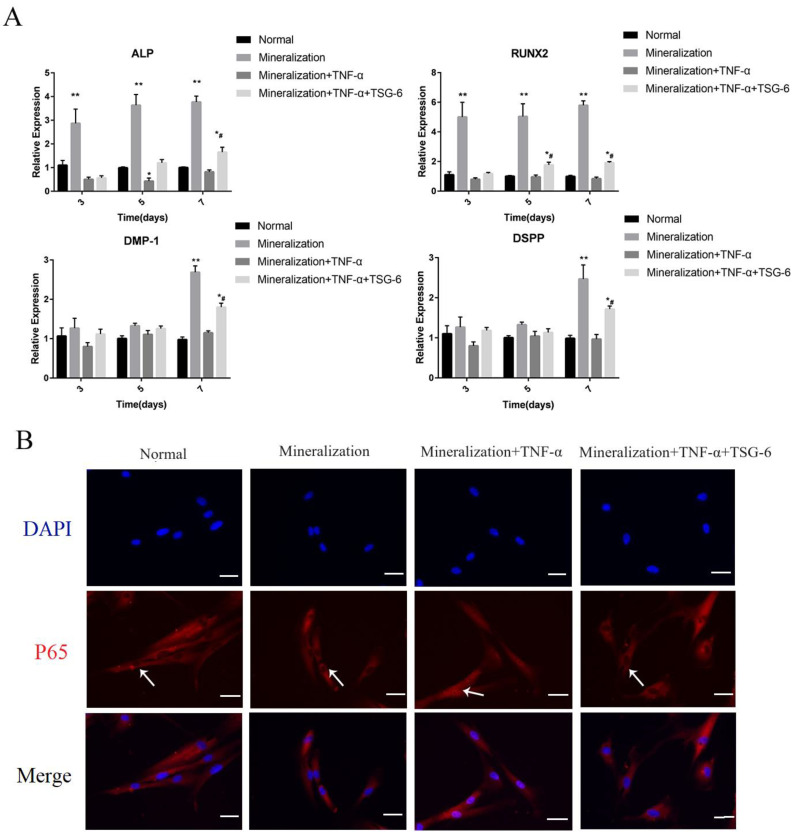

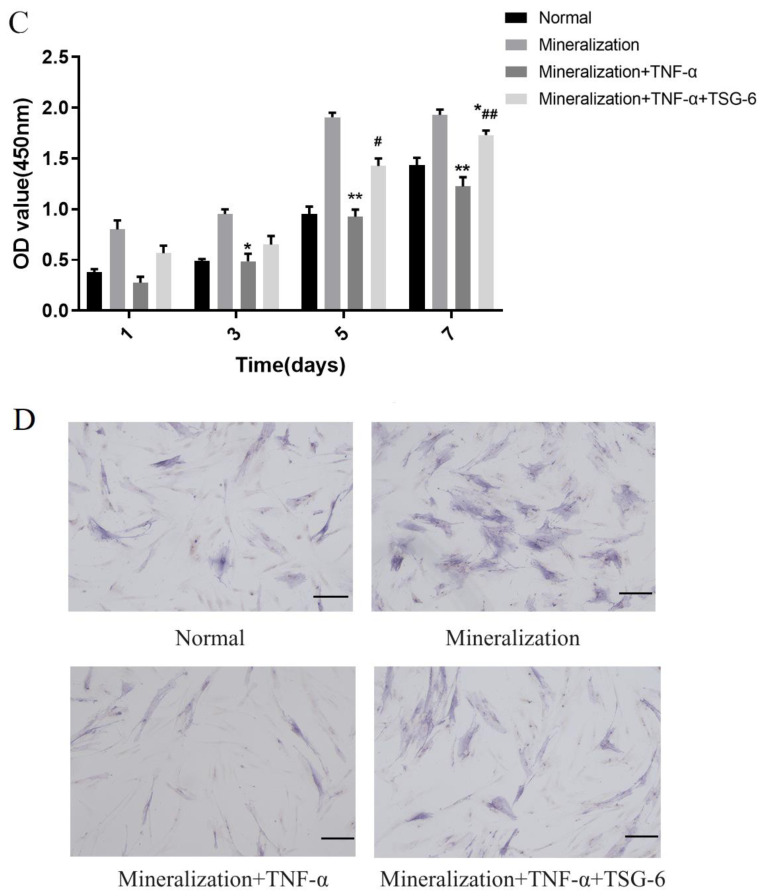
TSG-6 upregulated the mineralization ability of dental pulp stem cells in an inflammatory environment. Cells in the normal group were incubated in normal complete medium. Cells in the odonto/osteogenic-induction group were cultured with mineralization-inducing solution. Cells in the inflammation group (TNF-α group) were incubated with a mineralization-inducting solution containing 50 ng/mL TNF-α. Cells in the TSG-6 group were incubated in normal complete medium containing 20 ng/mL TSG-6 for 48 h, then were transferred into inflammation induction medium. (**A**) Odonto/osteoblast-related gene expression at the mRNA level. (**B**) The change in p65 nuclear translocation in each group. (**C**) Optical density (OD) assay was performed on days 1, 3, 5, and 7. (**D**) Alkaline phosphatase (ALP) assays were performed 3 days after induction. (* *p* < 0.05, ** *p* < 0.01, compared with the normal group; ^#^ *p* < 0.05, ^##^ *p* < 0.01, compared with the inflammation-induced group; * *p* < 0.05, ** *p* < 0.01, compared with the mineralization-induced group).

The mineralization-inducing solution promoted the odontogenic/osteogenic differentiation of the DPSCs, as evidenced by the increased expression levels of *ALP*, *RUNX2*, *DMP-1*, and *DSPP* at the mRNA level (Figure 2A). However, 50 ng/mL of TNF-α led to decreased levels of odontogenic/osteogenic-related gene expression, indicating that this concentration inhibited DPSC differentiation in vitro. Pretreatment of the DPSCs with TSG-6 partially restored their mineralization ability in the inflammation-inducing solution (Figure 2A). We observed NF-κB signaling pathway-related markers and found an increase in p65 nuclear translocation under inflammation-inducing conditions, indicating activation of the NF-κB signaling pathway. However, after TSG-6 pretreatment, the DPSCs no longer showed NF-κB signaling activation during inflammation, as p65 nuclear translocation was significantly inhibited (Figure 2B). 

A CCK-8 assay was used to observe changes in the proliferative capacity of the DPSCs (Figure 2C). From day 0 to 3, none of the groups showed significant differences in mineral content. On day 3, the inflammatory group showed a lower proliferation rate than the mineralized group. On day 5, the TSG-6 group exhibited a higher proliferation rate than the inflammatory group. These results suggested that the inflammatory environment was not conducive to the proliferation of the DPSCs and that TSG-6 treatment enhanced the proliferation of the DPSCs during inflammation. 

**Figure 3:**
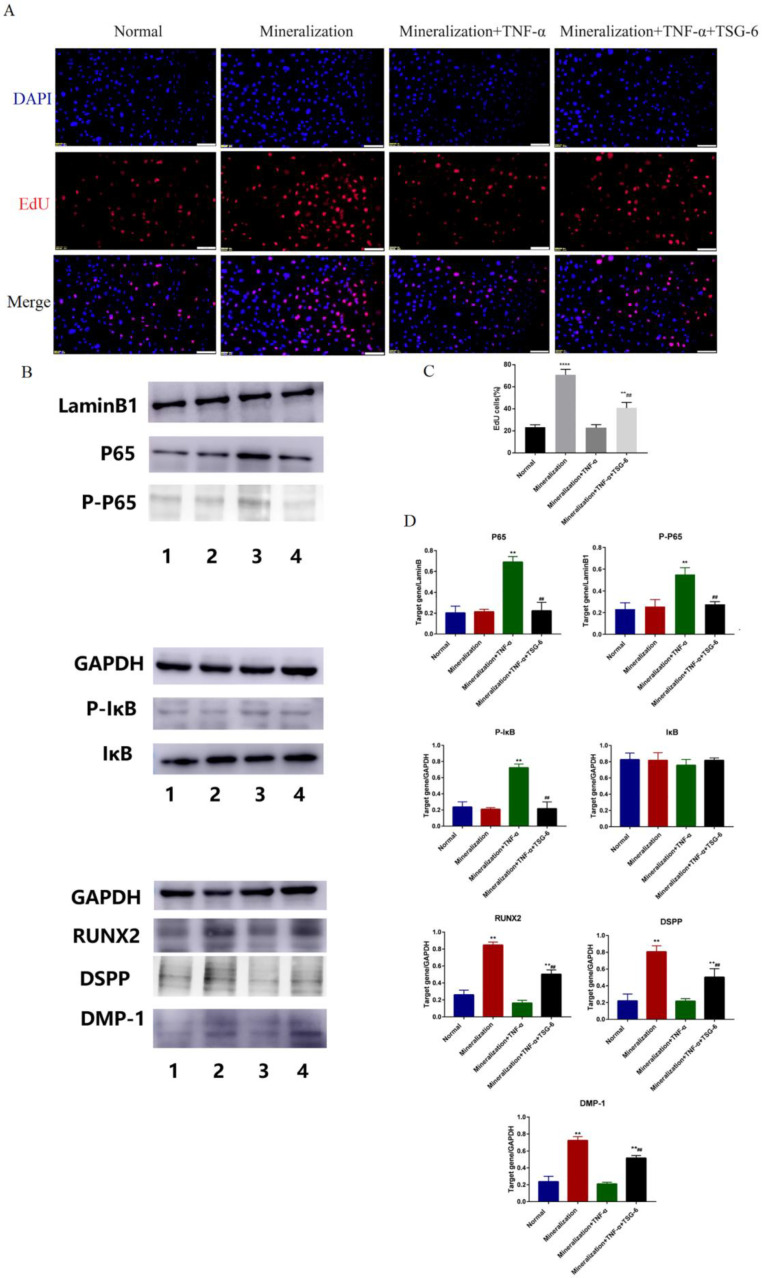
TSG-6 enhanced the odonto/osteoblast differentiation of dental pulp stem cells by inhibiting the nuclear factor kappa B signaling pathway. Dental pulp stem cells (DPSCs) were induced in different media as indicated. (**A**) 5-Ethynyl-2’-deoxyuridine (EdU) staining. (**B**) Expression levels of NF-κB pathway-related proteins, including p65, phosphorylated (p)-p65, IκB, and P-IκB, and odonto/osteogenic-related proteins RUNX2, DMP-1, and DSPP (1. Normal; 2. Mineralization; 3. Mineralization+TNF-α; 4. Mineralization+TNF-α+TSG-6). Original images can be found in Figure S1. (**C**,**D**) Statistical analysis results. (** *p* < 0.01, **** *p* < 0.0001, compared to the normal group; ^##^ *p* < 0.01, compared to the inflammation-induced group). Scale bars = 20 μm.

ALP activity is an important indicator of the odonto/osteogenic differentiation of DPSCs. In the inflammation-inducing solution, the ALP activity of the DPSCs was significantly reduced (Figure 2D). However, the DPSCs in the TSG-6 group exhibited significantly higher ALP activity than those in the inflammatory group, which was consistent with the real-time PCR results. We then observed the cellular activity of the DPSCs in each group using an EdU assay (Figure 3A). The mineralization-induced group exhibited the highest cellular activity, followed by the TSG-6 group, which had lower cellular activity than the other two groups, indicating that inflammation not only reduced the proliferative capacity of the DPSCs, but also inhibited their activity. This suggested that TSG-6 pre-treatment partially alleviated the inhibition of mineralization in an inflammatory environment.

#### 3.1.2. Exogenous TSG-6 Treatment Promoted the Mineralization of DPSCs by Inhibiting the NF-κB Signaling Pathway

TNF-α is a recognized agonist of the NF-κB signaling pathway. After induction for 48 h, Western blotting was performed to detect the expression levels of NF-κB pathway proteins in each DPSC group. Among the nuclear proteins, p65 and p-p65 expression levels increased in the inflammatory group, and p65 and p-p65 expression levels were not significantly different between the mineralized and TSG-6 groups and the normal group (Figure 3B), suggesting that the DPSCs did not exhibit excessive inflammatory signals after TSG-6 pretreatment. Among the cytoplasmic proteins, p-IκB expression levels were significantly higher in the inflammatory group, whereas no significant difference was seen in the remaining three groups (Figure 3B). 

Because IκB is phosphorylated after inflammatory stimulation, the p65-IκB complex disassociates and p65 enters the nucleus to activate downstream target genes. After 7 d of induction, Western blotting was performed to detect the expression levels of odonto/osteogenic-related proteins in each DPSC group (Figure 3B). RUNX2, DMP-1, and DSPP were used as indicators of mineralization. Compared to the normal group, only the mineralized and TSG-6 groups exhibited enhanced expression levels of mineralization-related proteins, suggesting that activation of the NF-κB signaling pathway reduced the odonto/osteogenic differentiation ability of the DPSCs. The TSG-6 group exhibited significantly higher levels of mineralization-related proteins than the inflammatory group, indicating that TSG-6 pretreatment enhanced the odonto/osteogenic differentiation of DPSCs in an inflammatory environment by inhibiting the NF-κB signaling pathway.

#### 3.1.3. CD44 was the “Switch” Whereby TSG-6 Inhibited the NF-κB Signaling Pathway and Enhanced the Mineralization of DPSCs

**Figure 4:**
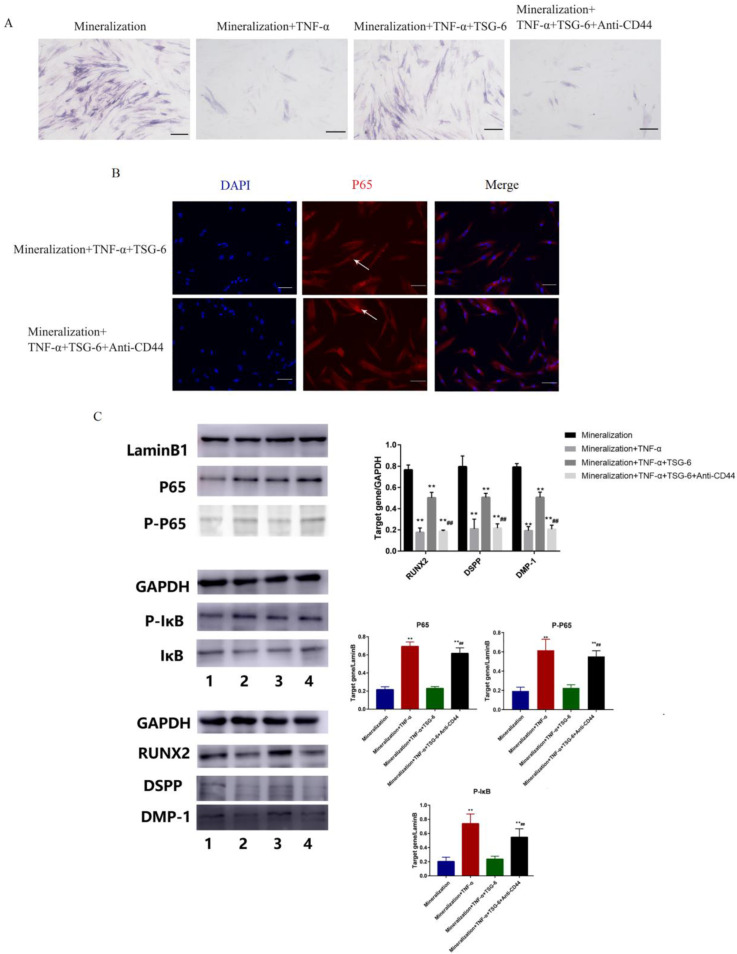
TSG-6 inhibited the nuclear factor kappa B signaling pathway in dental pulp stem cells via CD44. Cells in the odonto/osteogenic-induction group were cultured with mineralization-inducing solution. Cells in the TNF-α group were incubated with a mineralization-inducing solution containing 50 ng/mL TNF-α. Cells in the TSG-6 group were incubated in inflammation induction solution with TSG-6 pre-treatment. Cells in the anti-CD44 antibody group were incubated in inflammation induction solution with TSG-6 and anti-CD44 antibody pre-treatment. (**A**) ALP activity in each group. (**B**) p65 nuclear translocation. (**C**) Expression levels of NF-κB pathway-related proteins, including p65, p-p65, IκB, and P-IκB, and odonto/osteogenic-related proteins RUNX2, DMP-1, and DSPP. (** *p* < 0.01, compared to the odonto/osteogenic-induction group; ^##^ *p* < 0.01, compared to the TSG-6 group). Original images can be found in Figure S2. Scale bars = 20 μm.

We added an anti-CD44 antibody to DPSCs treated with TSG-6 to observe the changes in the NF-κB signaling pathway and odontogenic/osteogenic-related proteins. We first examined the changes in ALP activity in each group (Figure 4A). After the addition of the anti-CD44 antibody, the ability of TSG-6 to counteract the effects of inflammation was lost. Compared with the TSG-6 group, the anti-CD44 antibody group exhibited significantly lower ALP activity. Cellular immunofluorescence analysis revealed significantly higher levels of p65 and p-p65 nuclear translocation in the anti-CD44 antibody group (Figure 4B), indicating activation of the NF-κB signaling pathway in this group, and the inhibition of TSG-6 downregulated this signaling pathway. Subsequently, Western blotting was performed to detect the expression levels of NF-κB-pathway- and odontogenic/osteogenic-related proteins in each group (Figure 4C). Among the nuclear proteins, the inflammation and anti-CD44 antibody groups showed higher p65 expression levels. Among the plastid proteins, higher levels of p-IκB were observed in the inflammatory and anti-CD44 antibody groups, indicating that the inflammatory pathway was activated at the protein level. Levels of the mineralization marker proteins RUNX2, DMP-1, and DSPP were reduced in the anti-CD44 antibody group relative to their levels in the TSG-6 group, suggesting that CD44 is the switch for TSG-6 to counteract the effect of inflammation, and that TSG-6 interacts with CD44 on the cell membrane of DPSCs to affect the NF-κB signaling pathway, ultimately enhancing the odonto/osteogenic ability of DPSCs in an inflammatory environment.

#### 3.1.4. TSG-6 Was Beneficial for Subcutaneous Osteogenesis and Mandibular Bone Culture in an Inflammatory Environment

**Figure 5:**
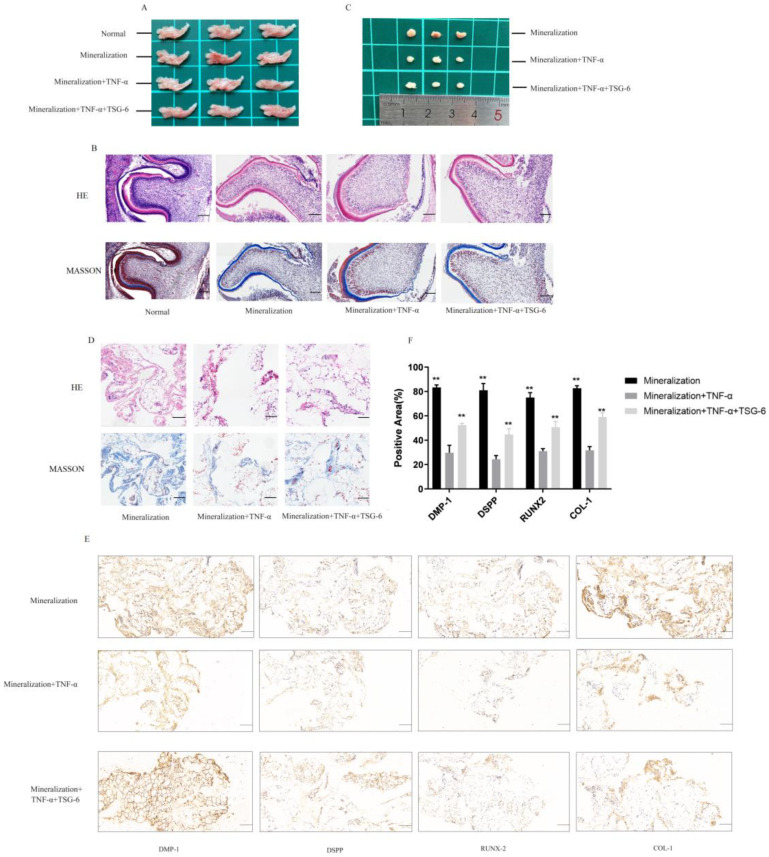
TSG-6 promoted the mineralization ability of dental pulp stem cells in an inflammatory environment in vivo. Sprague-Dawley (SD) rats were anesthetized and executed by isoflurane at the age of 2 days. The mandibles of the newborn rats were removed for in vitro culture for 10 days. DPSCs in the mineralized, inflammatory, and TSG-6 groups were induced in vitro for 7 days and then implanted into the backs of nude mice. The grafts were collected after 6 weeks. (**A**) A gross view of the rat mandibles. (**B**) Hematoxylin and eosin (HE) and Masson’s trichrome staining of the mandibles. (**C**) A gross view of the grafts. (**D**) HE and Masson’s trichrome staining of the grafts. Immunohistochemical staining results (**E**) and the bar graph (**F**) show odonto/osteogenic-related marker expression levels in the grafts. (** *p* < 0.01, compared with the inflammation-induced group). Scale bars = 20 μm.

Sprague-Dawley (SD) rats were anesthetized and sacrificed at two days old using isoflurane. The mandibles of the newborn rats were removed for in vitro culture (Figure 5A). Predentin appeared light pink upon HE staining and light blue upon Masson’s trichrome staining (Figure 5B). After 10 d, more predentin was deposited in the mineralized group than in the normal group. Dental embryos in the TSG-6 group showed a greater amount of predentin deposition than those in the inflammatory group, suggesting that TSG-6 treatment promotes dentin formation in mandibular culture experiments.

DPSCs in the mineralized, inflammatory, and TSG-6 groups were induced in vitro for seven days, implanted into the backs of nude mice, and the grafts were collected after six weeks. No significant changes were observed in the grafts of any group upon visual inspection (Figure 5C). HE and Masson’s trichrome staining were used to observe the structure and morphology of the grafts. The grafts in the mineralized group possessed more collagen fibers, which are the main components of pre-existing bone tissue (Figure 5D). After TSG-6 pretreatment, the grafts generated more collagen fibers in the inflammatory environment, indicating a greater propensity for osteogenesis. Immunohistochemical staining was used to assess the expression of odonto/osteogenesis-related proteins, including DMP-1, DSPP, RUNX2, and collagen I, in the grafts (Figure 5E). Grafts in the mineralized group exhibited higher levels of mineralization-related markers, while those in the inflammatory group exhibited lower expression levels of these markers. These results suggested that TSG-6 partially rescued the osteogenic differentiation of DPSCs in vivo and counteracted the effects of inflammation.

## 4. Discussion

Previous studies have shown that DPSCs play an important role in the treatment of endodontic diseases [30,31]. Due to the high prevalence of caries and pulpitis among dental patients, tooth repair and regeneration have significant clinical importance [32]. Severe tooth injury penetrates the enamel and dentin and stimulates the pulp to produce a limited natural repair process, resulting in the formation of new dentin-forming cells that give rise to new dentin [33]. The repair process is facilitated by the presence of undifferentiated cells in the dental pulp. The tooth has limited ability to repair damage; however, DPSCs can act as seed cells to replace damaged cells and promote their repair [34]. In addition, DPSCs can be easily obtained from a wide variety of sources and are used to restore vital pulp tissue removed by infection, rebuild periodontal ligaments lost due to periodontal disease, and generate complete or partial tooth structures to form biological implants [35]. 

**Figure 6:**
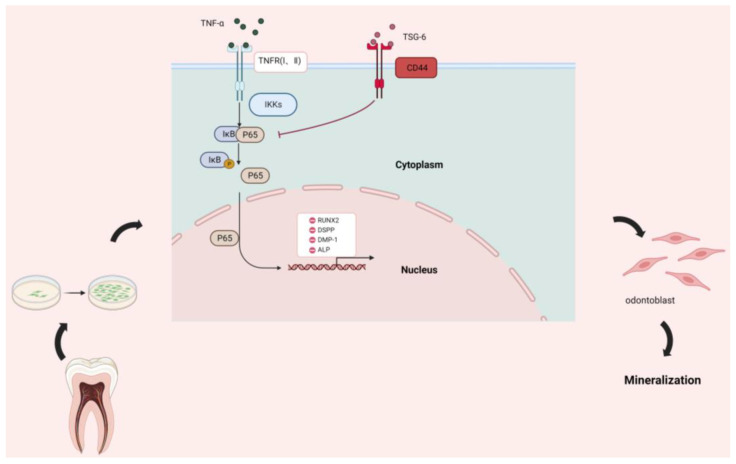
Summary. Pre-treatment of dental pulp stem cells (DPSCs) with tumor necrosis factor induced protein-6 (TSG-6) overcomes the differentiation inhibition induced by high concentrations of tumor necrosis factor-alpha (TNF-α). TSG-6 inhibits the nuclear factor kappa B (NF-κB) signaling pathway by binding to CD44 and drives the odonto/osteogenic differentiation of DPSCs.

Our findings confirmed that DPSCs are among the most potent seed cells for the treatment of endodontic diseases. By investigating methods to improve the odonto/osteogenic differentiation of DPSCs in an inflammatory environment, we found that exogenous administration of TSG-6 promoted the anti-inflammatory effects of DPSCs and partially restored their restorative function. We also determined that the mechanism of action of TSG-6 was attributable to its interaction with CD44 (Figure 6). We also demonstrated that TSG-6 inhibited the NF-κB signaling pathway in DPSCs. These findings revealed novel mechanisms by which DPSCs counteract the effects of inflammation.

Notably, the mineralization ability of MSCs is inhibited in a strongly inflammatory environment [36], and our experimental results, which involved simulating an inflammatory environment using TNF-α, supported this conclusion. In our study, the ability of DPSCs to differentiate into odontoblasts and produce restorative dentin was reduced in an inflammatory environment. However, pre-induction with exogenous TSG-6 promoted the mineralization of DPSCs, even in an inflammatory environment. 

TSG-6 is composed primarily of two modules, ΔCUB and ΔHA [37]. TSG-6 has multiple functions, including controlling stromal organization and the association of stromal molecules with cell surface receptors and extracellular signaling factors, such as chemokines [38]. A groundbreaking discovery regarding TSG-6 in the last few years is that it can mediate the immunomodulatory and tissue-protective properties of MSCs to some extent [39]. And mRNA expression of TSG-6 in cultured MSCs is significantly upregulated after TNF-α treatment. 

TSG-6 production is induced in response to inflammatory mediators such as IL-1, lipopolysaccharide, transforming growth factor beta (TGF-β), and TNF-α [40]. TSG-6 has also been studied in mineralized tissue and found to be associated with arthritis in humans and is produced by chondrocytes and synovial cells [41]. TSG-6 is produced in large quantities in the synovial fluid of patients with rheumatoid arthritis, osteoarthritis, and other arthritic conditions [42]. However, it is unclear whether TSG-6 production is beneficial in musculoskeletal pathology.

DPSCs also secrete a certain amount of TSG-6 upon inflammatory stimulation; however, their odonto/osteogenic differentiation capacity is reduced. The reason for this finding remains unclear. We speculate that local secretion is insufficient or that TSG-6 does not act directly on DPSCs. In this study, the “rescue function” of TSG-6 was lost after an anti-CD44 antibody was added. Moreover, sustained induction of TSG-6 did not play a role in promoting DPSC mineralization, and the effects of an inflammatory environment were reversed only when DPSCs were pretreated with TSG-6. Although the detailed mechanism is still unclear, one possible explanation is that TSG-6 acting alone can cause the down-regulation of NF-κB; therefore, it does not cause the inhibition of downstream-related differentiation pathways when DPSCs are subjected to inflammatory stimulation. 

CD44 also plays an important role in the differentiation of dental embryos and is associated with hyaluronic acid, which is the structural basis of its interaction with TSG-6 [43,44]. We found that pre-administration of TSG-6 reduced the inhibition of odonto/osteogenic processes induced by high concentrations of TNF-α. In conjunction with the results of experiments in nude mice and mandible culture experiments, our findings support the potential of TSG-6 for the treatment of pulpitis. In addition, we demonstrated that the effect of TSG-6 depends on inhibition of the NF-κB signaling pathway in DPSCs. 

These results are consistent with those reported previously. Both physiological and pathological remodeling of skeletal homeostasis are associated with NF-κB signaling, and the over-activation of NF-κB leads to osteoporosis and inflammatory bone loss [45,46]. In addition, NF-κB is involved in osteoclast activation. NF-κB is also involved in the resorption of dental hard tissues and the inhibition of odontoblast differentiation. However, our research still has some limitations. Using TNF- α only cannot fully reproduce the pulpitis microenvironment. Further evidence is needed to verify the clinical therapeutic effect of TSG-6 on pulpitis. In addition, further research is needed to provide methods for the local application of TSG-6.

In conclusion, we found that pre-treatment with TSG-6 rescued the differentiation inhibition of DPSCs induced by high concentrations of TNF-α. TSG-6 inhibited the NF-κB signaling pathway by binding to CD44 and induced the odonto/osteogenic differentiation of DPSCs. These findings have implications for the development of new therapeutic methods using DPSCs and offer additional possibilities for pulpitis treatment and tooth regeneration.

## Data Availability

Data is unavailable due to privacy.

## Supplementary Materials

The following supporting information can be downloaded at: https://www.mdpi.com/article/10.3390/biom14030368/s1, Figure S1: Original western blots for Figure 3; Figure S2: Original western blots for Figure 4.
