# Supplementary figures and images for: TSG-6 Inhibits the NF-κB Signaling Pathway and Promotes the Odontogenic Differentiation of Dental Pulp Stem Cells via CD44 in an Inflammatory Environment
Source: Biomolecules. 2024 Mar 19;14(3):368. doi: 10.3390/biom14030368 (PMC10968114; doi:10.3390/biom14030368)

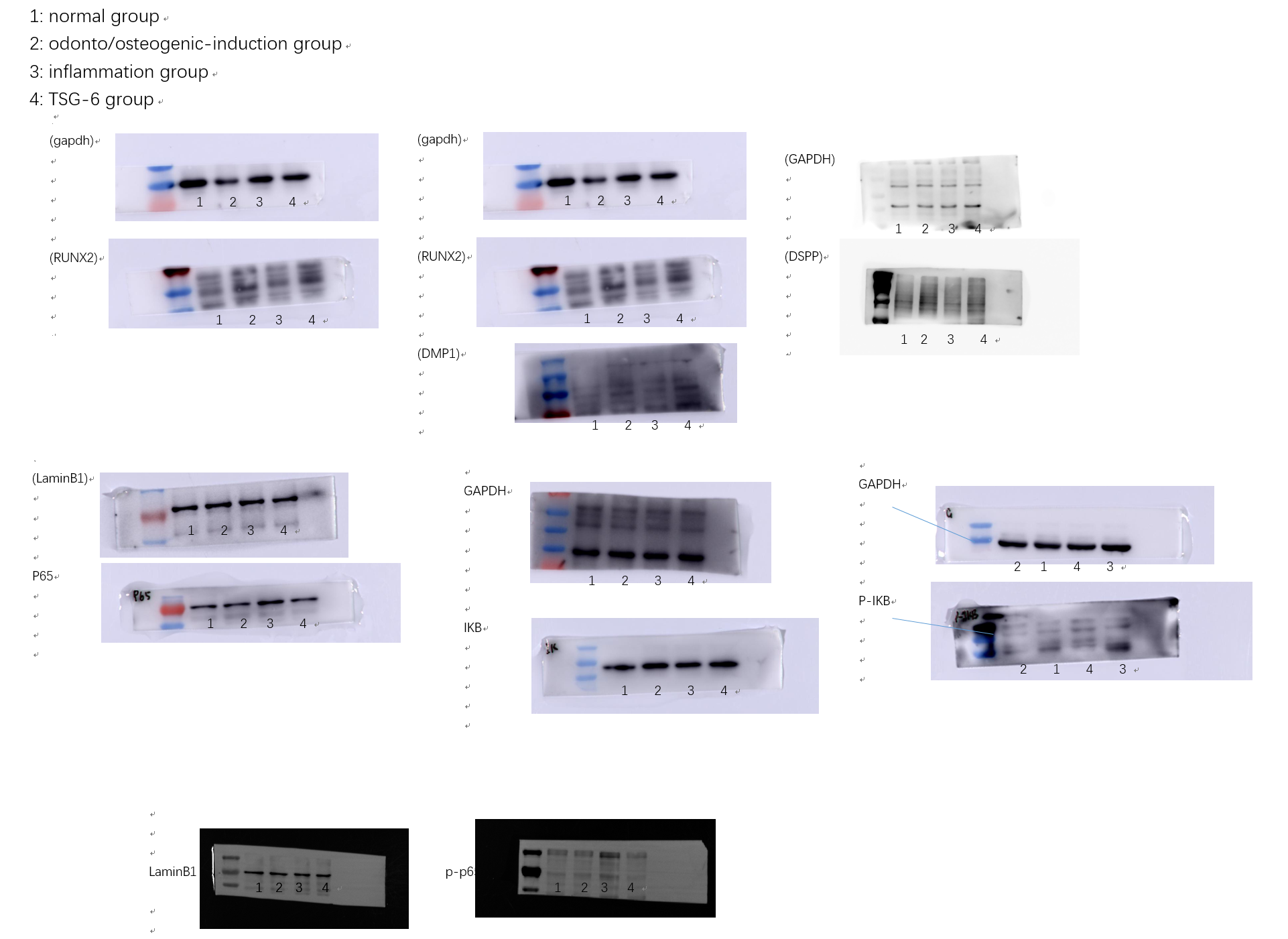

Supplement: Supplementary file 1 [file biomolecules-14-00368-s001.zip › FigureS1.tif]

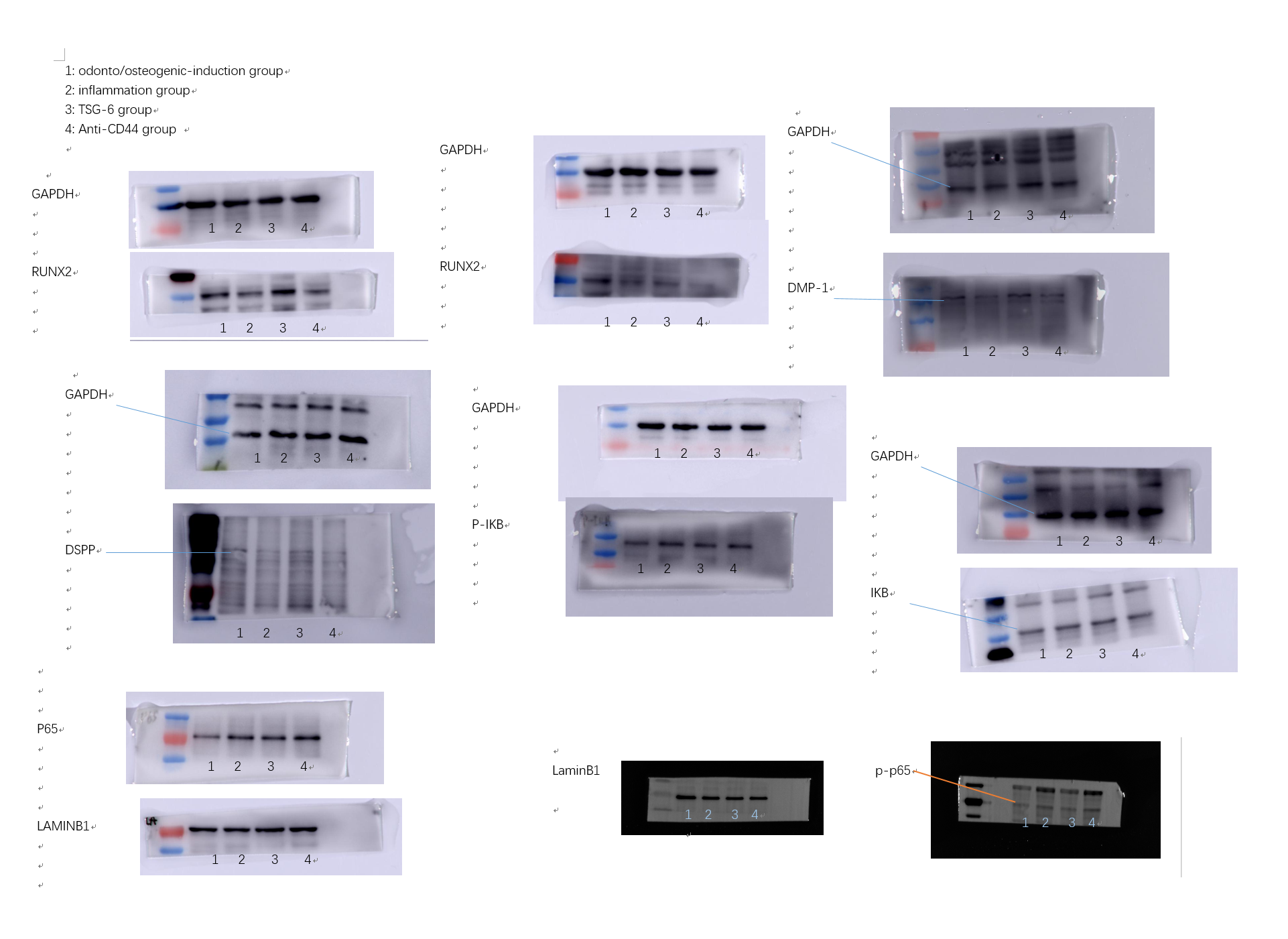

Supplement: Supplementary file 1 [file biomolecules-14-00368-s001.zip › figureS2.tif]
